# FLPP: A Federated-Learning-Based Scheme for Privacy Protection in Mobile Edge Computing

**DOI:** 10.3390/e25111551

**Published:** 2023-11-16

**Authors:** Zhimo Cheng, Xinsheng Ji, Wei You, Yi Bai, Yunjie Chen, Xiaogang Qin

**Affiliations:** 1 Department of Next-Generation Mobile Communication and Cyber Space Security, Information Engineering University, Zhengzhou 450002, China; ndscjxs@126.com (X.J.); youwei1102@163.com (W.Y.); baiyi@mail.ndsc.com.cn (Y.B.); chenyunjie2009@qq.com (Y.C.); 15639750306@163.com (X.Q.); 2 Purple Mountain Laboratories, Nanjing 211111, China

**Keywords:** mobile edge computing, privacy protection, federated learning, differential privacy, differential evolutionary

## Abstract

Data sharing and analyzing among different devices in mobile edge computing is valuable for social innovation and development. The limitation to the achievement of this goal is the data privacy risk. Therefore, existing studies mainly focus on enhancing the data privacy-protection capability. On the one hand, direct data leakage is avoided through federated learning by converting raw data into model parameters for transmission. On the other hand, the security of federated learning is further strengthened by privacy-protection techniques to defend against inference attack. However, privacy-protection techniques may reduce the training accuracy of the data while improving the security. Particularly, trading off data security and accuracy is a major challenge in dynamic mobile edge computing scenarios. To address this issue, we propose a federated-learning-based privacy-protection scheme, FLPP. Then, we build a layered adaptive differential privacy model to dynamically adjust the privacy-protection level in different situations. Finally, we design a differential evolutionary algorithm to derive the most suitable privacy-protection policy for achieving the optimal overall performance. The simulation results show that FLPP has an advantage of 8∼34% in overall performance. This demonstrates that our scheme can enable data to be shared securely and accurately.

## 1. Introduction

With the rise of mobile edge computing (MEC), massive amounts of data are being generated by a wide variety of sensors, controllers and smart devices [1]. In the era of the Internet of Everything, data utilization is key to enabling innovation, driving growth and solving our major challenges [2]. By data mining, we can reveal the hidden patterns, trends and correlations. This information helps us make optimal decisions, for instance, the precise diagnosis and treatment of diseases in the medical field, or the optimization of traffic flow and resource allocation in urban planning. Evidently, the integrated utilization of data can bring great value and benefits [3].

However, it is often difficult to derive value from the data of a single user. More user data needs to be involved in the analysis and refinement to get comprehensive information  [4]. In traditional centralized machine learning, data is often stored centrally in a centralized server. This leads to the isolated data island effect, i.e., data cannot be fully utilized and shared. Meanwhile, data privacy protection has become a key issue because of the centralization of users’ sensitive personal data [5]. Data from mobile devices generally should not be shared with others in mobile edge computing scenarios. Therefore, breaking the isolated data island and ensuring data privacy is a current issue [6].

Federated learning (FL) [7], as a new technology paradigm based on cryptography and machine learning, can achieve information mining without local data. It can unite data distributed in different mobile devices and train them into a unified global model with more comprehensive information. Thus, it solves the problem of isolated data islands. The clients and server interact with data information through the model parameters without sharing the original data, improving their data privacy [8].

However, federated learning also leads to several security and privacy risks [9]. One of the main threats is model inference attack. Although communication is channeled through the model parameters, Zhu et al. [10] revealed that exchanged model parameters may also leak private information about the training data. They demonstrated that the original training data, including image and text data, can be inferred from the gradients. This poses a new challenge for data privacy-preserving techniques based on federated learning.

To address the above problems, we propose FLPP: a federated-learning-based scheme for privacy protection in MEC. FLPP enables data centralization across multiple devices while protecting data privacy in mobile edge computing scenarios. The main contributions of this paper are as follows.

(1) Targeting heterogeneous data, we present a federated-learning-based scheme for privacy protection in MEC. The scheme can improve the accuracy of training by adjusting the weights of its model parameters according to the amount of different users’ data. In addition, a differential privacy technique is implemented by adding noise to the model parameters so as to protect the privacy of user data.
(2) To achieve flexible adjustment of differential privacy, we build a layered adaptive differential privacy model. During each epoch of training, different levels of noise can be added to cope with the requirements under various conditions.
(3) Due to the higher privacy level, the model training is influenced by noise resulting in lower accuracy. In order to trade off the accuracy and security of the model, we customize a differential evolution algorithm to derive the optimal policy to achieve the best overall performance.

The rest of the paper is organized as follows. Section 2 discusses the related work. In Section 3, we present the threat model and formulate the data privacy issues. Section 4 depicts the details of the FLPP scheme. Section 5 evaluates our work with existing methods. Finally, Section 6 concludes the study.

## 2. Related Work

Existing studies enhance the security of federated learning by combining with a variety of privacy-protection techniques, mainly including homomorphic encryption (HE), secure multi-party computation (SMPC) and differential privacy (DP) [11]. Extensive research demonstrates that the combination of federated learning with these privacy-protection techniques can provide sufficiently strong security.

Fang et al. [12] proposed a multi-party privacy-preserving machine learning framework, named PFMLP, based partially on HE and federated learning. Training accuracy is achieved while also improving the training efficiency. Xu et al. [13] proposed a privacy-protection scheme to apply HE in IoT-FL scenarios, which is highly adaptable with current IoT architectures. Zhang et al. [14] propose a privacy-enhanced federated-learning (PEFL) scheme to protect the gradients over an untrusted server. This is mainly enabled by encrypting participants’ local gradients with a Paillier homomorphic cryptosystem. The HE approach can improve the security of federated learning, although it causes a large computation load. This poses a challenge to the limited computability of devices in mobile edge computing scenarios.

Kalapaaking et al. [15] proposed a federated-learning framework that combines SMPC-based aggregation and Encrypted Inference methods. This framework maintains data and model privacy. Houda et al. [16] presented a novel framework, called MiTFed, that allows multiple software defined network (SDN) domains to collaboratively build a global intrusion detection model without sharing their sensitive datasets. The scheme incorporates SMPC techniques to securely aggregate local model updates. Sotthiwat et al. [17] propose to encrypt a critical part of model parameters (gradients) to prevent deep leakage from gradient attacks. Fereidooni et al. [18] present SAFELearn, a generic design for efficient private FL systems that protects against inference attacks. In addition, recent studies [19,20,21] on secret sharing techniques as a kind of SMPC also hopefully enable federated learning and data sharing security. The above studies implement the secure construction of models but cannot afford the communication overhead of a large number of participants.

The differential privacy technique is a good way to avoid the computation load and communication overhead. Wang et al. [22] proposed a collaborative filtering algorithm recommendation system based on federated learning and end–edge–cloud computing. The exposure of private data was further prevented by adding Laplace noise to the training model through DP technology. Wei et al. [23] proposed a novel DP-based framework, NbAFL, in which artificial noise is added to parameters at the clients’ side before aggregating. The strategy for achieving the optimal performance and privacy level is performed by selecting the number of clients participating in FL. Zhao et al. [24] propose an anonymous and privacy-preserving federated-learning scheme for the mining of industrial big data, which leverages differential privacy on shared parameters. They also test the effect of different privacy levels on accuracy. Adnan et al. [25] conduct a case study of applying a differentially private federated-learning framework for analysis of histopathology images, the largest and perhaps most complex medical images. Their work indicates that differentially private federated learning is a viable and reliable framework for the collaborative development of machine learning models in medical image analysis. However, the DP privacy level of these works is fixed so it cannot adapt to the dynamically changing sets of participating aggregation clients. In particular, non-IID data distribution with fixed privacy level may slow down the speed of FL model training to reach the anticipated accuracy.

In summary, the DP technique with adjustable privacy levels is clearly more suitable for privacy protection for federated learning in mobile edge computing. To this end, we propose FLPP, a privacy-protection scheme based on federated learning to adaptively determine a privacy level strategy, aiming to jointly optimize the accuracy and security of the training model.

## 3. System Model and Problem Formulation

**Figure 1:**
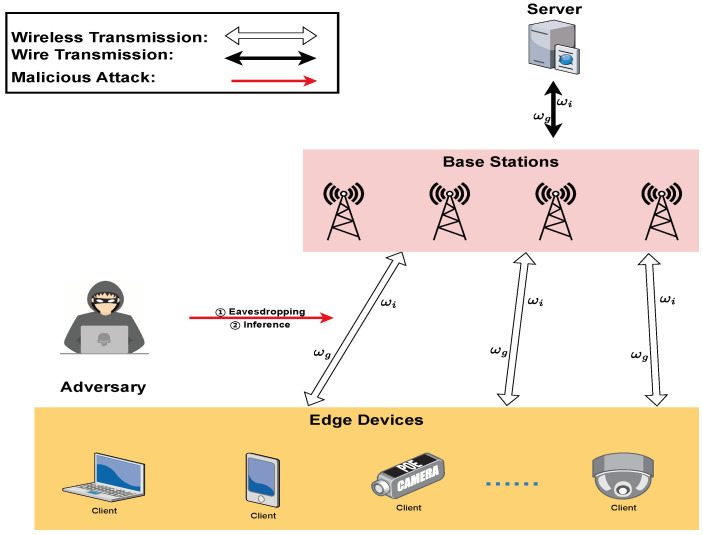
Overview of federated-learning-based MEC system.

In this section, we elaborate a federated-learning-based MEC system, as shown in Figure 1. Firstly, we propose a data privacy threat model for the MEC system. In order to achieve data centralization and privacy protection across multiple devices, we present mathematical models for data protection, parameter protection and problem statement.

### 3.1. Threat Model

In the system, a trusted MEC server acquires data from the mobile devices under its range and classifies the data for aggregation. These mobile devices can offload data to adjacent base stations via wireless transmission. And BSs are wired connected with the MEC server, forming a fundamental mobile edge computing network.

We assume that there are M={1,2,⋯,m} mobile devices connected to this MEC server in the scenario. At each slot *t*, the mobile devices have data $D=\{d_{1},d_{2},\cdots ,d_{m}\}$ to be transmitted to the MEC server for data aggregation. At the same time, the mobile devices can also request the aggregation results from the MEC server. In this case, some data privacy issues may occur [26].

(1) Eavesdropping: Also called sniffing or snooping attack, eavesdropping refers to picking up a transmitted packet sent over the network. The edge nodes directly offloaded will be vulnerable to malicious attacks against the data itself, causing privacy leakage.
(2) Membership Inference Attacks: As the name denotes, an inference attack is a way to infer training data details. Attackers obtain the gradient information of the aggregation process by eavesdropping or other methods. Then, this information can be used to infer more valuable intelligence.

### 3.2. Data Protection Model

To address the above risks, we employ federated learning for data protection, which is a machine learning framework. It can achieve the target of joint modeling by transmitting parameters without local data from multiple participants. The FL task flow mainly includes local training, parameter upload, model aggregation and parameter distribution [27]. The total dataset associated with the task is given by

(1) $$D=\sum_{m=1}^{M}d_{m}.$$

- **step 1** Local Training: Each node trains the model locally according to its own data after the MEC server distributes the initial model to each edge node.
  Gradient descent of client *i* can be expressed as
  (2) $$g_{i}=F_{i}\left(\omega_{g}\right),$$
  where $\omega_{g}$ is the distributed model parameter and $F_{i}$ denotes the loss function of client *i*.
  The updated model parameter of client *i* can be calculated by
  (3) $$\omega_{i}=\omega_{g}-\eta g_{i},$$
  where η is the learning rate.
- **step 2** Model Uploading: The participating nodes upload the model parameters obtained from local training to the MEC server.
- **step 3** Model Aggregating: The MEC server securely aggregates the uploaded model parameters to get the updated global model parameter.
  Each aggregated weight is related to the size of the node dataset and the updated global model parameter can be expressed as
  (4) $${\omega_{g}}^{\prime }=\sum_{i\in I}\frac{d_{i}}{\sum_{i\in I}d_{i}}\omega_{i},$$
  where *I* stands for the set of participating clients.
- **step 4** Model Broadcasting: The server broadcasts the updated global model parameter to each edge node and starts a new round of training.
  (5) $$\omega_{g}={\omega_{g}}^{\prime }.$$

The outcome of the training can be evaluated by using accuracy as a criterion. The accuracy is defined as the test accuracy of the aggregated global model on the test dataset which can be represented by the loss functions as

(6) $$A=F(d_{test},\omega_{g}),$$

where $d_{test}$ is the test dataset.

However, there are still some potential risks in achieving data protection by federated learning. Scholars have found that exchanged model parameters may also disclose private information about the training data [28,29].

### 3.3. Model Parameter Protection Model

Although the node uploads a model parameter rather than the data themselves under the structure of federated learning, it is undeniable that the model gradients are trained directly from the private data of the participants. Therefore, there is a possibility of privacy leakage by inference on the gradient information. Differential privacy uses the randomized response method to ensure that the dataset is always affected by a single record below a certain threshold when outputting information. Consequently, third parties cannot determine changes in the data itself based on differences in the output.

For an arbitrary query function *f*, *D* and $D^{\prime }$ are adjacent datasets that differ by at most one record. The sensitivity is determined by comparing the maximum change value of the two datasets, denoted as

(7) $$\Delta f=\max_{D,D^{\prime }}{∥f\left(D\right)-f\left(D^{\prime }\right)∥}_{t},$$

where t represents the norm.

To reduce the sensitivity, we can use a differential privacy algorithm to make the output of querying two datasets similar, when there is a randomized algorithm Q satisfying

(8) $$Pr\left[Q\left(D\right)=O\right]\leq e^{\varepsilon }Pr\left[Q\left(D^{\prime }\right)=O\right],$$

where *O* is the output of the algorithm *Q*. In this case, we consider that the algorithm satisfies differential privacy.

Obviously, the smaller the ε, the stronger the privacy protection. However, the strength of privacy protection decreases as the data availability increases.

Laplace noise as a common noise mechanism can satisfy ε− difference privacy. Laplace noise serves to add a noise of the same scale as the model parameters to the actual output. The probability of the amount of noise added is positively correlated with the λ value. This helps to hide the real model parameters. However, adding noise will inevitably affect the accuracy of the model training because of fluctuations in the actual parameters. We should ensure that the privacy-protection technique has a certain strength of protection. Meanwhile, the final training results are not interfered with too much by noise. We add Laplace noise to the users’ model parameters at each round of aggregation, which can be expressed as

(9) $$\omega_{i}^{\prime }=\omega_{i}+\left[Lap_{1}\left(\lambda \right),Lap_{2}\left(\lambda \right),\ldots ,Lap_{n}\left(\lambda \right)\right],$$

(10) $$\lambda =\frac{\Delta_{f}}{\varepsilon },$$

where *n* indicates the round of aggregation. This fuzzification prevents malicious participants from inferring the user’s private data while enabling the model training function.

In addition, this approach achieves model parameter protection without increasing the security overhead of the client compared to homomorphic encryption and secure multi-party computation.

### 3.4. Problem Statement

Both accuracy and security of federated learning need to be considered in the data aggregation process. However, the implementation of gradient protection changes the original gradient information, which affects the accuracy. Moreover, higher privacy-protection strength leads to lower accuracy. Therefore, the goal of optimizing privacy protection is

(11a) $$P:\underset{D,j}{\arg\;\max}\;A_{J}+\frac{\sum_{j=1}^{J}\lambda_{j}}{J\lambda_{max}},$$

(11b) $$s.t.\;\;\;\;A_{J}\geq 0.7,$$

(11c)     j∈J,

(11d) $$\;\;\;\;\lambda_{min}\leq \lambda_{j}\leq \lambda_{max}.$$

Constraint (11b) indicates that the final accuracy should exceed 0.7. Constraint (11c) indicates that the round number *j* is within the total round number *J* of federated-learning training. Constraint (11d) defines the available range of the privacy level in each round.

The challenge in solving problem *P* is to trade off global accuracy and security. Also, the selection of parameters for each communication round is crucial to the final result. Therefore, we propose a differential evolution-based algorithm to solve problem *P* and formulate the privacy-protection scheme FLPP. On the one hand, the differential evolution algorithm has better global search capability and higher convergence speed. On the other hand, the algorithm has low complexity and is easy to implement so that it does not impose a computational load on the system.

## 4. FLPP Scheme

**Figure 2:**
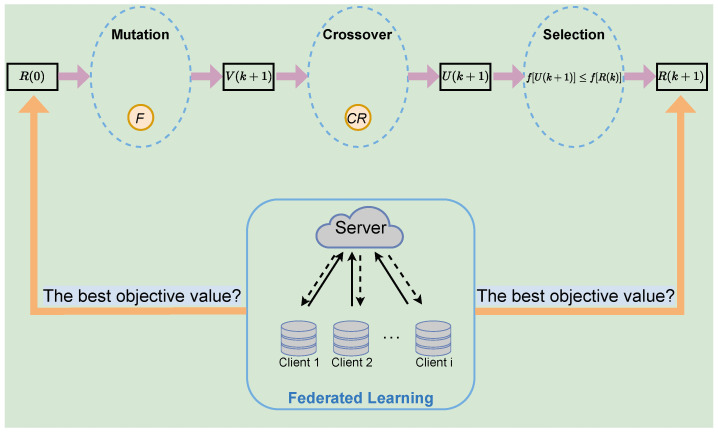
Overview of FLPP scheme.

The details of the FLPP scheme are presented in this section, as shown in Figure 2. This scheme first uses federated learning to convert raw data into a model parameter for transmission to avoid data leakage directly. Then, a differential privacy technique is employed to defend against inference attacks targeting federated learning, which enhances the privacy-protection capability of the scheme. Finally, the scheme can adaptively determine the privacy level policy in order to jointly optimize the accuracy and security of the training model. This effectively improves the dynamic privacy protection of user data in mobile scenarios, while ensuring data availability. The FLPP scheme is organized into two parts: the federated-learning algorithm and the privacy-protection optimization algorithm. Further descriptions are as follows.

### 4.1. Algorithmic Framework of Federated Learning

With the emergence of isolated data islands and increasing concern for personal privacy protection, the mobile edge computing scenario requires a distributed machine learning framework to support it. As one of the contributions of this paper, we propose a privacy-protection scheme based on federated learning. On the one hand, federated learning can aggregate data from multiple independently distributed edge nodes, effectively solving the problem of isolated data islands. On the other hand, the mode of local training and gradient interaction ensures that data does not leave the local area. The traditional data privacy dilemma is tackled. The entire federated-learning algorithm framework is shown in Algorithm 1.
**Algorithm 1** Federated Learning
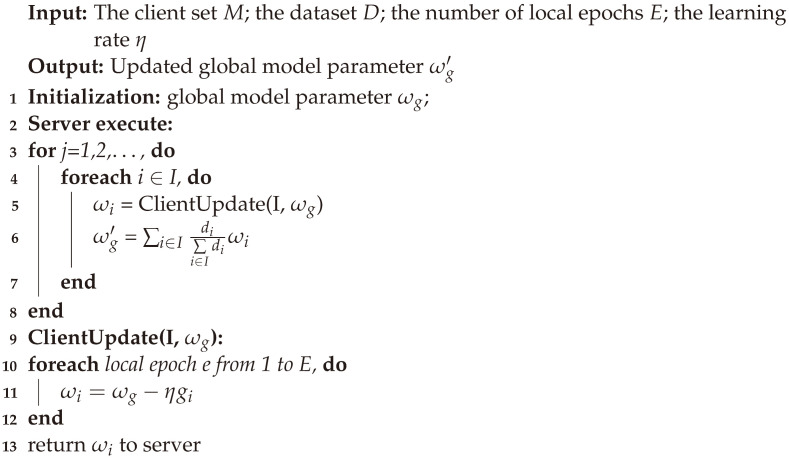


   Federated learning is the basis of the FLPP scheme. After receiving the initial model from the MEC server, the edge nodes train through the local data separately and independently. Then, the results of the model training are evaluated by the loss function. The loss function is related to the predicted and real values of the nodes on the sample dataset, which can be defined as

(12) $$F\left(\omega \right)=\sum_{i\in I}\frac{d_{i}}{\sum_{i\in I}d_{i}}H_{i}\left(p,q\right).$$

We employ the cross-entropy function to assist in calculating the loss function, which can be expressed as

(13) $$H\left(p,q\right)=-\sum_{j\in J}p\left(j\right)\log q\left(j\right),$$

where *p* denotes the true value, *q* represents the predicted value and H(p,q) stands for the cross-entropy loss. In order to obtain the optimal model parameters, the loss function is required to be minimized, such that

(14) $$\omega =\arg\;\min F_{i}\left(\omega \right).$$

In this paper, the approach for updating the model parameters is Adam, combining the advantages of Momentum and AdaGrad. It can both accommodate sparse gradients and mitigate gradient oscillation. The process of the Adam algorithm can be expressed as

(15) $$m_{t}=\beta_{1}m_{t-1}+\left(1-\beta_{1}\right)g_{t},$$

(16) $$v_{t}=\beta_{2}v_{t-1}+\left(1-\beta_{2}\right)g_{t}^{2},$$

(17) $$\hat{m_{t}}=\frac{m_{t}}{1-\beta_{1}^{t}},$$

(18) $$\hat{v_{t}}=\frac{v_{t}}{1-\beta_{2}^{t}},$$

(19) $$\theta_{t+1}=\theta_{t}-\eta \frac{1}{\sqrt{\hat{v_{t}}}+\epsilon }\hat{m_{t}}.$$

Until obtaining the optimal local model parameter, the client transmits the parameter back to the server.

### 4.2. Privacy-Protection Optimization Algorithm

Due to the performance inadequacy of federated-learning privacy protection, additional privacy techniques are needed to enhance protection. We guarantee differential privacy protection by injecting Laplacian noise of equal scale into the optimal model parameter.

(20) $$\omega_{i}^{\prime }=\omega_{i}+N\left(0,\lambda \right),$$

where N is the Laplace noise. The probability density function of the Laplace distribution is expressed as

(21) $$p\left(x\right)=\frac{1}{2\lambda }e^{-\frac{\left|x\right|}{\lambda }},$$

where λ controls scaling of the function. It can be seen that the larger lambda, the more noise is added so that the security is increased. However, the accuracy of the model will decrease at the same time.

Meanwhile, FL tasks require multiple rounds of iterative training to complete. Each round of training has different participating nodes, resulting in different amounts of data and different data types. It is impossible to adapt the training rounds to different data distributions by still using a fixed λ. In particular, an inappropriate λ would interfere with the speed of global model building and decrease the accuracy rate.

Therefore, we propose a multi-layer adaptive differential privacy-protection scheme. We set Λ=[0.1,0.2,…,0.5] to meet the privacy-protection requirements in different situations. The parameter can be adaptively adjusted at each training round according to the demand of model training accuracy and security.

The FLPP scheme employs the differential evolutionary algorithm (DE) to obtain the optimal policy. DE is an intelligent optimizing algorithm inspired by biological evolution, which is based on the genetic algorithm (GA) [30]. The DE algorithm achieves a heuristic search for complex search spaces by simulating the genetic process, which can eventually find the global optimal solution with higher probability. It also supports parallel computation, which shortens the search time. The algorithm firstly randomly generates privacy-protection policies as initial populations. Through mutation, crossover and selection, the optimal privacy-protection policy is finally obtained. The privacy-protection optimization algorithm is described specifically as below and shown in Algorithm 2.
**Algorithm 2** Differential Evolution
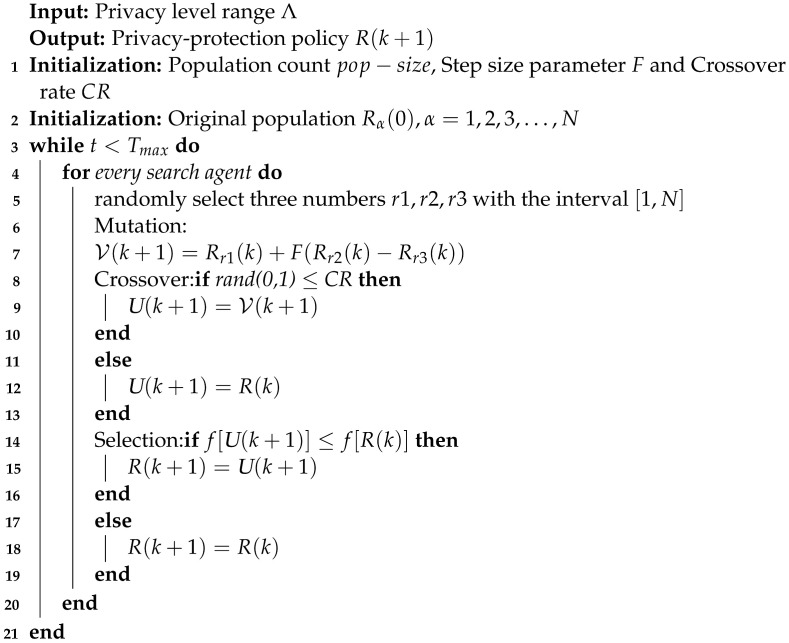


**Chromosome and Fitness Function**: In DE, each individual is defined by a chromosome, implying that an individual’s chromosome is a part of the solution to problem **P**. In this problem **P**, the chromosome of each individual is a privacy policy, which consists of the privacy level for *J* training rounds. To reduce the number of variables to be optimized, we transfer $\left\{\lambda_{1},\lambda_{2},\ldots ,\lambda_{J}\right\}$ to *R*, which is denoted by

(22) $$R_{\alpha }\left(0\right)={\left[\lambda_{1},\lambda_{2},\ldots ,\lambda_{J}\right]}^{T},\alpha =\left\{1,2,3,\ldots ,N\right\}$$

where λ is the privacy level in each training round.

**Initialization**: The initial population (i.e., initialization in Algorithm 2) is generated randomly in this framework, but under constraints 11e and 11d of Problem **P**. The original population’s genes are created as follows:

(23) $$\lambda_{j}\left(0\right)=random.sample\left(\Lambda ,1\right),j\in J$$

where random.sample(Λ,1) is a generator function outputting a random value in the range Λ.

**Mutation**: DE achieves individual mutation through a difference policy. We employ a commonly used difference policy where two different individuals in the population are randomly selected, and their vector differences are scaled and synthesized with the individuals to be mutated:

(24) $$V\left(k+1\right)=R_{r1}\left(k\right)+F\left(R_{r2}\left(k\right)-R_{r3}\left(k\right)\right)$$

where r1, r2 and r3 are three random numbers with the interval [1,N], *F* is a deterministic constant representing the scaling factor and *k* denotes the number of generations.

**Crossover**: The purpose of this step is to randomly select individuals. Since differential evolution is also a randomized algorithm, so the crossover is performed by

(25) $$U\left(k+1\right)=\left\{\begin{array}{cc} V(k+1) & \;\;if\;rand(0,1)\leq CR\\ R(k) & otherwise \end{array}\right.$$

where CR is the crossover probability; namely, new individuals are generated with a random probability.

**Selection**: DE adopts a greedy strategy to select an optimal individual among the results of crossover to continue evolution.

(26) $$R\left(k+1\right)=\left\{\begin{array}{cc} U(k+1) & if\;f[U(k+1)]\leq f[R(k)]\\ R(k) & otherwise, \end{array}\right.$$

where

(27) $$f=A_{J}+\frac{\sum_{j=1}^{J}\lambda_{j}}{J\lambda_{max}}.$$

This indicates that the overall performance of the algorithm is determined by both accuracy and security.

## 5. Simulation and Discussion

In this section, we simulate a scenario containing a MEC server and 50 mobile devices. The server coordinates all mobile devices in the scenario for federated-learning training to obtain an accurate data model. The experiments are performed on python 3.7.13 and pytorch 1.13.1 under the Ubuntu 18.04 operating system. We conduct experiments on the standard MNIST dataset for handwritten digit recognition, consisting of 60,000 training examples and 10,000 testing examples. Each example is a 28 × 28 size gray-level image. We randomly assign the dataset to each client and ensure that a certain number of clients participate in training in each round. The relevant simulation parameters are shown in Table 1.

In order to realize data sharing and privacy protection, this proposal employs accuracy and security as performance metrics. Accuracy is obtained from Equation (6). Security is obtained from the privacy level set by the scheme. Accuracy is a prerequisite for data to be shared correctly. Security is the key for data not to be leaked. The overall performance consists of accuracy and security, which can reflect the effect of the scheme more comprehensively. Therefore, we analyze and evaluate it in terms of training, security, accuracy and overall performance. Under different training rounds, this research proposal compares with other existing research proposals [23,31,32]. These studies protect the federated-learning model with fixed privacy level parameters, such as NbAFL. In contrast to fixed privacy level parameters, the FLPP scheme employs adaptively adjustable privacy level parameters. Based on their studies, the comparison proposals are privacy protected by different fixed privacy level parameters within the range of the defined privacy level, which are the actual intent of the NbAFL. Therefore, we believe that the distinction between the FLPP scheme and these existing proposals can be shown in equivalent experimental scenarios. The specific evaluation results are as follows.

### 5.1. Performance of Training

**Figure 3:**
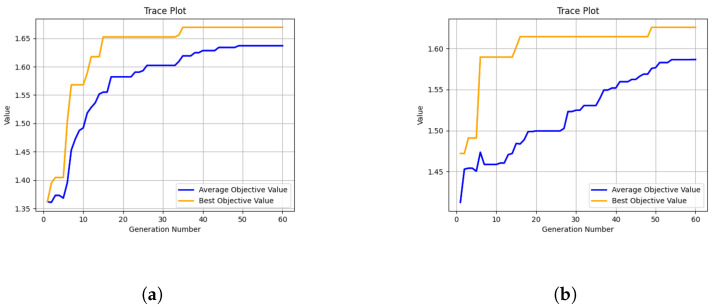
Convergence curves of DE. (**a**) Five rounds of training. (**b**) Ten rounds of training.

Figure 3 represents the convergence curves of the DE algorithm for 5 and 10 training rounds. The horizontal axis denotes the number of population evolution generations and the vertical axis indicates the objective value. It can be observed that the DE algorithm converges well and obtains the best objective value at the 35th and 49th generation, respectively. Meanwhile, the average objective value also converges and has the maximum value at the 49th and 54th generation, respectively. This shows that the algorithm evolves correctly and the best objective value obtained is the global optimal solution.

### 5.2. Overall Performance

**Figure 4:**
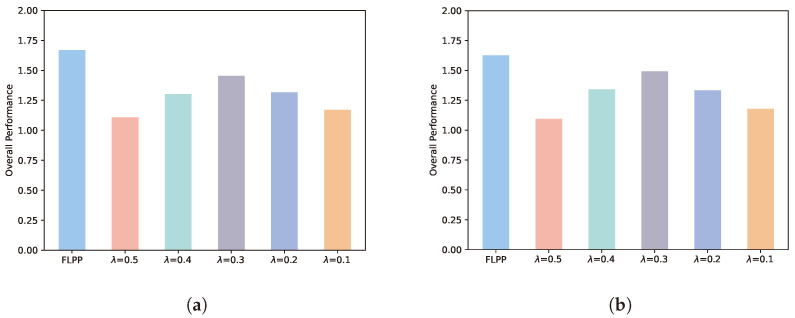
Overall performance. (**a**) Five rounds of training. (**b**) Ten rounds of training.

Figure 4 illustrates the comparison between the FLPP and five fixed privacy level schemes. We denote the objective value of the scheme, i.e., the overall performance, by Equation (27). It can obviously be seen that the overall performance of FLPP is better than other fixed schemes at different numbers of training rounds. This indicates that FLPP can achieve the optimal decision according to different numbers of training rounds. At five rounds of training, FLPP has an advantage of 34%, 22%, 13%, 21% and 30% over the fixed privacy level schemes, respectively. At 10 rounds of training, the overall performance of FLPP improves by 33%, 18%, 8%, 18% and 28%, respectively, over the comparison schemes. We can conclude from the results as below. A fixed privacy level cannot effectively cope with the changeable training environment. The FLPP scheme can dynamically adjust the privacy level according to the actual situation of each training round to achieve the best training outcome. In addition, the increase in training rounds can narrow the gap between the comparison schemes and FLPP. However, the number of training rounds is limited in mobile edge computing scenarios. Mobile devices cannot stay in the range of one server for a long time to participate in federated learning. Therefore the FLPP scheme is suitable for this research scenario.

### 5.3. Accuracy Performance

**Figure 5:**
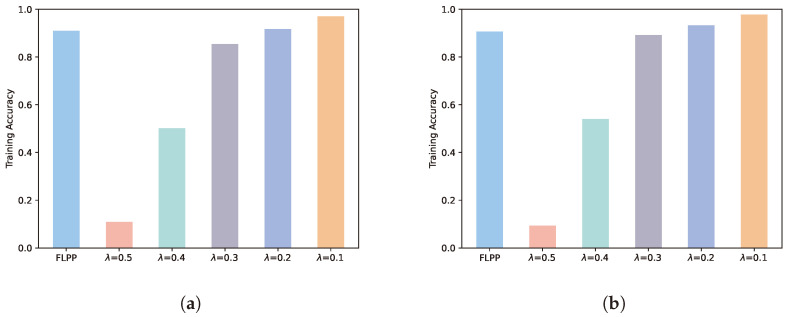
Accuracy performance. (**a**) Five rounds of training. (**b**) Ten rounds of training.

Figure 5 depicts the performance of the global model accuracy obtained by federated learning for the FLPP scheme versus the other comparison schemes. At five rounds of training, the accuracy of the FLPP scheme increases by 88%, 45% and 6% over λ = 0.5, λ = 0.4 and λ = 0.3 while decreasing by 1% and 7% over λ = 0.2 and λ = 0.1. At 10 rounds of training, the FLPP scheme has 90%, 40% and 2% improvement, and 3% and 8% reduction in accuracy from the same comparison scheme. In addition to normal training loss, the main factor affecting model accuracy is the privacy level of the added noise. In case the privacy level is increased, the model accuracy decreases accordingly. Figure 5 demonstrates that the accuracy of the FLPP scheme is greatly improved compared to the high privacy level scheme. Compared to the low privacy level scheme, the accuracy margin of the FLPP scheme is not significant. This indicates that the FLPP scheme is sufficient to train global models with good accuracy.

### 5.4. Security Performance

**Figure 6:**
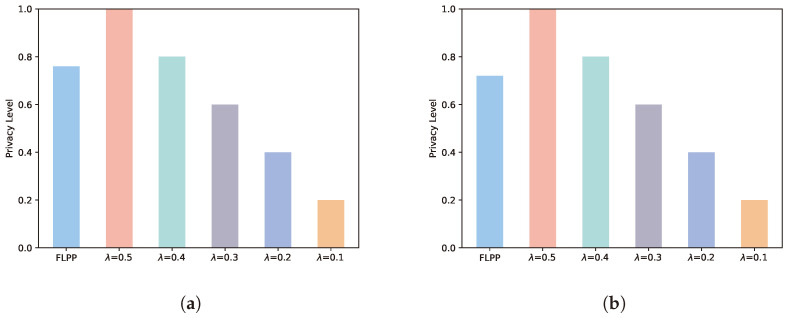
Security performance. (**a**) Five rounds of training. (**b**) Ten rounds of training.

Figure 6 demonstrates the comparison of different schemes in terms of their security performance. The security performance is defined as the ratio of the sum of the privacy levels used in each round to the sum of the maximum privacy levels employed in each round. A greater value indicates a better security performance. At five rounds of training, the FLPP scheme reduces its security by 32% and 5% compared to λ = 0.5 and λ = 0.4 as well as increases it by 21%, 47% and 74% compared to λ = 0.3, λ = 0.2 and λ = 0.1. Similarly, the FLPP scheme decreases security by 39% and 11% over λ= 0.5 and λ = 0.4 while improving it by 16%, 44% and 72% over λ=0.3, λ = 0.2 and λ = 0.1 at 10 rounds of training. Merely considering the security, FLPP is not the optimal solution. This is due to the fact that the λ = 0.5 and λ = 0.4 schemes only take into account the security and neglect the accuracy of the model training.

## 6. Conclusions

In order to break the isolated data island and prevent data privacy leakage, this paper proposes a federated-learning-based privacy-protection scheme FLPP in mobile edge computing scenarios. In the dynamic training situation, this scheme takes into account the accuracy and security of the data model. First, we establish a training framework of federated learning to convert raw data into model parameters. Afterwards, we employ the differential privacy technique to protect the privacy of model parameters. Finally, the DE algorithm dynamically adjusts the parameters of the privacy-protection level according to actual situations and further obtains the optimal privacy policy. Simulation results show that the FLPP scheme has the best overall performance by integrating accuracy and security. This provides an effective solution for data privacy protection in mobile edge computing scenarios.

## Figures and Tables

**Table 1:** Simulation parameters.

Parameter	Value
Number of clients	50
Data volume of clients	[1, 60,000]
Number of participating clients	[4, 10]
Privacy level range	[0.1, 0.5]
Learning rate	0.005
Number of local epochs	10
Crossover rate	0.7
Step size parameter	0.5

## Data Availability

Data are contained within the article.

## Abbreviations

The following abbreviations are used in this manuscript:

Notation	Meaning
M	Set of mobile devices
η	Learning rate
D	Set of data volume of mobile devices
$d_{test}$	Test dataset
I	Set of participating clients
ε	Privacy budget
J	Total training rounds
Δf	Sensitivity of dataset
$\omega_{g}$	Global model parameter
λ	Privacy level
$\omega_{i}$	Model parameter of client *i*
A	Training accuracy
